# Ultrashort echo-time MRI as a substitute to CT for skull aberration correction in transcranial focused ultrasound: i*n vitro c*omparison on human calvaria

**DOI:** 10.1186/2050-5736-3-S1-P12

**Published:** 2015-06-30

**Authors:** Jean-Francois Aubry, Matt Eames, John Snell, Wilson Miller

**Affiliations:** 1 University of Virginia, Charlottesville, Virginia, United States; 2 Focused Ultrasound Foundation, Charlottesville, Virginia, United States

## Background/introduction

Clinical transcranial MR-guided focused ultrasound (TcMRgFUS) brain treatment systems compensate for skull-induced beam aberrations by adjusting the phase and amplitude of individual ultrasound transducer elements. These corrections are currently calculated based on a pre-acquired CT scan of the patient’s head. The purpose of the work presented here is to demonstrate the feasibility of using ultrashort echo-time (UTE) MRI instead of CT to calculate and apply aberration corrections on a clinical TcMRgFUS system.

## Methods

Phantom experiments were performed in three *ex vivo* human skulls filled with tissue mimicking hydrogel. Each skull phantom was imaged with both CT and UTE MRI. The MR images were then segmented into “skull” and “not-skull” pixels using a computationally efficient, threshold-based algorithm, and the resulting three-dimensional binary skull map was converted into a series of two-dimensional virtual CT images. Each skull was mounted in the head transducer of a clinical TcMRgFUS system (ExAblate Neuro, Insightec, Israel), and transcranial sonications were performed using a power setting of approximately 750 Acoustic Watts at several different target locations within the electronic steering range of the transducer. Each target location was sonicated three times: once using aberration corrections calculated from the actual CT scan, once using corrections calculated from the MRI-derived virtual CT scan, and once without applying any aberration correction. MR thermometry was performed in conjunction with each 10-second sonication, and the highest single-pixel temperature rise and surrounding-pixel mean were recorded for each sonication.

## Results and conclusions

**Figure 1:**
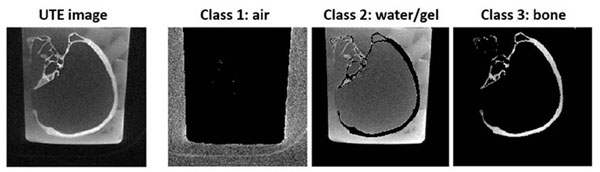
Representative UTE MR image and segmentation results from skull #3. For imaging, the skull was immersed in a bucket of water. The segmentation algorithm assigned each image voxel to one of three classes: (1) air, (2) water/gelatin, (3) bone.

**Figure 2:**
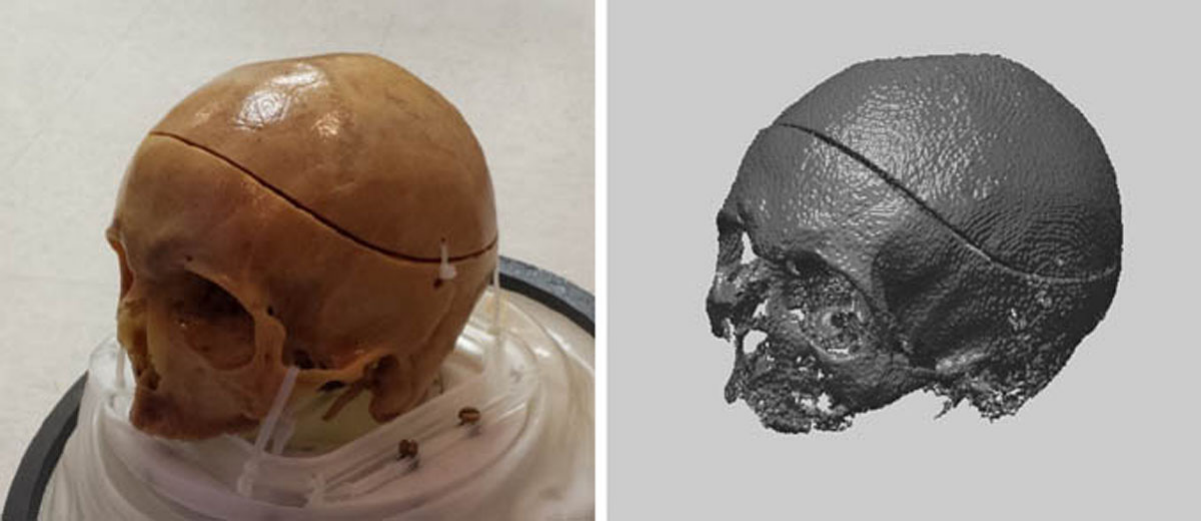
Photograph of one of the skull phantoms (#2), and a 3D surface rendering generated from the MR-derived bone map.

**Figure 3:**
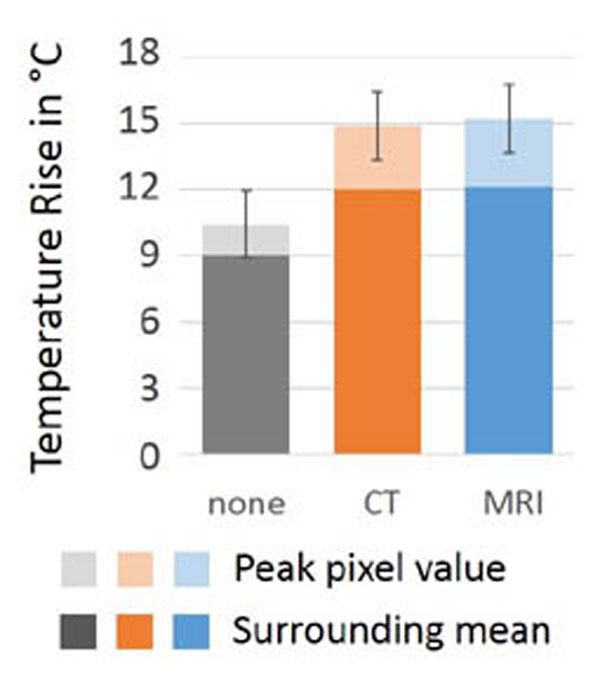
Graphical summary of sonication results. Each bar represents the average temperature rise of all sonications performed in all skulls using the same aberration correction method (none, CT-based, or MR-based).

Fig. 1 shows a UTE MR image and segmentation results from one of the skull phantoms. Fig. 2 shows a photograph of another skull phantom along with a 3D surface rendering generated from the binary bone map. The sonication results are summarized in Fig. 3. The measured temperature rises were ~45% larger for aberration-corrected sonications than for non-corrected sonications. This improvement was highly significant (p < 10–4). The difference between the single-pixel peak temperature rise and the surrounding pixel mean, which reflects the sharpness of the thermal focus, was also significantly larger for aberration-corrected sonications. There was no significant difference between the sonication results achieved using CT-based and MR-based aberration correction.

